# Editorial: Understanding cognitive differences across cultures: Integrating neuroscience and cultural psychology

**DOI:** 10.3389/fpsyg.2022.1041734

**Published:** 2022-09-28

**Authors:** Tachia Chin, Francesco Caputo, Chien-Liang Lin, Fengpei Hu

**Affiliations:** ^1^School of Management, Zhejiang University of Technology, Hangzhou, China; ^2^Department of Economics, Management, and Institutions, University of Naples Federico II, Naples, Italy; ^3^College of Science and Technology, Ningbo University, Ningbo, China

**Keywords:** cognitive differences, neuroscience, cultural psychology, cross-cultural studies, cross-cultural differences

## Introduction

Cultural psychology studies address how cultural factors affect human cognition and behavior (Amanzio et al., 2018; Calabrese et al., 2018; Adams et al., 2022; Del Giudice et al., in press), while neuroscience research provides profound insights into explaining the interplay of humans' brain systems with their attitudes and behaviors (Kenning and Plassmann, 2008; Amodio, 2014; Cascio et al., 2015). Obviously, the two disciplines show a high level of relevance, particularly in the domains elucidating how the underlying causal attributional processes of human mental programming and brain neural activity affect individual, organizational, and societal outcomes.

Numerous studies in cultural psychology have identified, examined, and interpreted cross-cultural variances in human cognition at different levels of analysis (Chentsova-Dutton, 2020; Chin et al., 2022; Lin et al., 2022). The renowned individualism–collectivism and holistic–analytic mental paradigms (Hofstede et al., 2010; Cleeremans, 2014; Hu et al., 2020) are good examples. Westerners tend to view the world as a composition of independent objects with a stronger desire for personal autonomy and victory, while Easterners often make holistic assumptions in favor of achieving balance and harmony in competing demands. However, despite the considerable cross-cultural variations discussed, the results remain controversial.

To fill the abovementioned gap, some scholars have advanced the integration of cultural psychology with neuroscience theories and methods, whereby cross-cultural comparisons can be elucidated through a more scientific lens. Unlike traditional behavioral measures, neuroscientific approaches provide synchronous, more objective observation of the neural processing of the brain. Nevertheless, given the infancy, complexity, and lack of cost efficiency of using neuroscience methods, hitherto limited evidence has been found, and more work needs to be done.

Taking together the foregoing arguments, this Research Topic aims to call for interdisciplinary studies with broader, multidisciplinary theoretical underpinnings or methodologies at the intersection of cultural psychology and neuroscience. To encourage and allow for more innovative submissions, we define “culture” in a broader way, encouraging authors to consider all levels of analysis of cultural differences. Fortunately, we are very pleased to claim that a wide range of fascinating articles have been received and published. We classified the 22 selected articles into five categories based on the methodology used and the key arguments discussed, whereby the main findings and perspectives are summarized below.

## Empirical studies on cultural impacts on human cognition and behavior

The first part contains 11 published articles that adopt frequently used empirical methods (i.e., qualitative, quantitative, or mixed approaches) in the areas of psychology to address cultural impacts on human cognition, perception, and behavior. Among these, the majority of articles underscore the uniqueness of Chinese cultural values and their influence on a variety of attitudinal and behavioral outcomes, which are elaborated as follows. Along with the rapid advancement of digital and big data technologies, Liu T. et al. investigate the application of face recognition technology in China; their research provides abundant practical implications, especially about the vital importance of enhancing Chinese users' trust in digital technology for reducing their concerns about privacy leaks. Using the Chinese cultural context as a research setting, Peng et al. point out the imperative to developing organizational emotional capability by demonstrating how the positive relationship between managers' psychological capital and employees' safety behavior is mediated by organizational emotional capability; their work indicates the key role of managers in promoting employee safety behaviors. In an attempt to address subcultural differences in China, Yu D. et al. report the negative impact of psychological distance on inter-group reciprocity and the negative effects of relationship-divisive and innovation-divisive faultiness on reciprocity within and between subgroups; their findings imply that, practically, firms' decision-making process for establishing effective technological innovation networks should take into account the selecting of suitable partners in the first place. Yu F. et al. demonstrate the East–West differences in the neural responses of executive directors to external pay gaps; their findings show that the positive effect of perceived compensation fairness of executives on their innovative motivation is more pronounced in the regions engrained with Confucian culture than those in Western culture.

Two papers address the effects of cultural values on online purchasing behavior. Incorporating Confucian values into the stimulus-organism-response (SOR) theory, Gao et al. explore the mechanisms of atmospheric cues and sales promotion in e-commerce live streaming on impulsive buying behavior and the mediating effects of the Zhong Yong thinking style and the emotions of online consumers on the abovementioned associations. Chen F. et al. unveil the East–West cultural differences about the effects of focal and alternative identities on the intent to purchase products; their results show that unlike Western consumers who prefer to buy products that fit with their primed identity, the purchase behavior of Chinese consumers is also largely influenced by the accessibility of the alternative identity.

There are three articles linking cultural differences to leadership. Shen and Lei employ a grounded study methodology to examine the negative effects of three main leadership characteristics (i.e., psychological, behavioral, and ability) on followers' counterproductive work behavior in the Chinese cultural context. From the perspective of social cognition, Meng et al.  investigate the influence of a Chinese culturally grounded leadership style, namely authoritarian–benevolent ambidextrous leadership, on employee innovative behavior; based on a qualitative methodology, their work provides some interesting, probably context-specific results for future research to dig deeper. Using the Chinese cultural context as a backdrop, Chen and Zhang assume the interrelations between mindful agency, metacognitive ability, and self-leadership; their results suggest the mediating effect of metacognitive ability on the relationship between mindful agency and self-leadership. Based on the data collected from Chinese multinational companies, Chen K et al.  find that the interactions of cross-cultural variances between the home and host countries and between the emerging and developed markets are significantly related to the learning mechanisms of cross-border merge and acquisition (M&A); their findings highlight the vital importance of cross-cultural understanding.

The last paper of the first part is written by Assens-Serral et al. who translate and validate a widely used English organizational culture assessment scale into a Spanish version; they empirically test the applicability of this scale in a Spanish context, while some difficulties in transferring the “*ad hoc* factor” have also been addressed. Their research provides fresh ideas for assessing the validity of mature scales/measures in culturally diverse contexts.

## Systematic reviews for identifying hot topics in cross-cultural studies

The second part includes four review papers. Di et al. employ the Web of Science (WoS) database to collect the co-citation of keywords in “cultural psychology,” “cross-cultural communication,” “neuroscience,” and “social media” and thereby conduct a bibliometric analysis; their results show an emerging trend of integrating multiple perspectives to develop future studies on cross-cultural communication through social media and in a virtual form. Xu et al. perform a bibliometric analysis in the field of cultural neuroscience from 2008 to 2021 based on the WoS database; this paper provides a holistic picture of the development trajectory of cultural neuroscience studies. Kang and Su perform a systematic review on the amazing link between digital reality technologies and creative and cultural industries beyond borders; in particular, they highlighted the importance of leveraging digital reality technologies to break cultural barriers and transform static cultural heritage exhibits into more appealing, engaging, and enjoyable experiences. Chen J. et al. conduct a systematic review with a bibliometric analysis on volunteer motivation from 2000 to 2021 and thereby identify the historical development of relevant studies; the unique value of this article lies in its comparative analysis of how cross-cultural differences between China and the United States affect volunteer motivation. In contrast to American volunteers motivated by individualistic values, Chinese volunteers believe in collectivist values.

## Reconceptualizing culture-related terms through a neuroscientific lens

In the third part, there are two perspective articles focusing on the reconceptualization of culture-related terms from an unconventional neuroscientific angle. Chen H. et al. expand the existing literature of multinational companies by reconceptualizing the internalization of firms to exploring the neural responses of top management to cultural diversity from a neuroeconomic angle; their study proposes that—through a neuroscientific lens—the internationalization of firms is mainly built upon the cultural identity and cognitive preference of their executives rather than upon the market determinants, as indicated by the theories of classical economics. Built upon a traditional aesthetic triad consisting of three dimensions (sensor-motor, knowledge-meaning, and emotion evaluation), Xie et al. address how the aesthetic cognitions of people with diverse cultures may differ in interpreting the sensory properties of nature (i.e., the architecture of the hotel in this case); their research also suggests that it is feasible to assess the aesthetic performance of an architecture masterpiece by observing its fluency, complexity, and naturalistic patterns.

## The role of learning culture during COVID-19

The fourth part collects two papers that demonstrate the role of learning culture in different contexts amid the pandemic. Liu H-L. et al. discuss how the learning mechanism can be affected by multiple psychological factors; they propose an educational learning system that relies on adaptive-feedback emotional computing technology to identify and interpret learners' emotional signals. With a similar focus on educational learning during the pandemic, Mo et al. place particular emphasis on investigating the impacts of cultural factors on the relationships between teachers and students, as well as the teaching modes and the students' learning motivation and styles; their findings show significant variances in individuals' cognition toward learning and teaching between Chinese and Western cultures.

## A linguistic aspect of cross-cultural differences

The fifth part contains three articles that explain cross-cultural differences from a linguistic angle. Chen Q. et al. explore the neurocognitive mechanisms in the English translation of Chinese poetry; based on a textual analysis of the first-person points of view and their immersive experience, their findings offer novel insights into understanding how the human brain acts as a critical neurotransmitter to the source text in translation. Canes trino et al. deem knowledge sharing a complex language-based activity in cross-border research collaborations, whereby they shed light on the vital role of linguistic abilities in facilitating knowledge sharing among participants within international university research teams. This research provides valuable implications for multinational research teams in achieving knowledge exchange and creation. Zhong and Liu provide potent theoretical underpinnings and novel explanations about how Chinese people conceptualize time and create time interval words; based on this, they conclude that the event-based metonymy conceptualization of time can enhance our understanding of the uniqueness of Chinese modes of thinking and its dynamic influence on human cognition and perception of the reality of the world.

## Conclusion

Overall, the 22 selected articles for our Research Topic (RT) cover a wide range of subjects, themes, and research domains with the use of multifaceted methodologies and theoretical grounds. Although the majority of the published articles come from China, we can still see quite a few international collaborations in authorship and affiliations. This partly echoes the aim of our RI to encourage cross-cultural communication and understanding by attracting collaborative research that transcends cultures.

As noted earlier, we categorize our RI into five sections. The first section, entitled “Empirical studies about cultural impacts on human cognition and behavior,” shows the importance of contextual dynamics in determining the influence of culture. The second section, entitled “Systematic reviews for identifying hot topics in cross-cultural studies,” provides a more holistic picture of current trends and future directions. The third section, entitled “Reconceptualizing culture-related terms through a neuroscientific lens,” helps justify the vital need for integrating cultural psychology and neuroscience to make sense of things in a cross-cultural setting. The fourth section, entitled “The role of learning culture during the COVID-19,” indicates the significance of learning in a highly uncertain environment. The fifth section, entitled “A linguistic aspect to cross-cultural differences,” implies that our deep culture may be reflected in the languages we use because we are unconsciously competent in our language.

Taking a close look at the five categories of the selected studies above, it is obvious that manifold consequences and causes of cultures on human cognition and behavior have been discussed; however, their effects may change under different circumstances. Viewed from this angle, our RT indeed adds unique value to the literature by enhancing the depth and breadth of relevant research. Given that heterogeneous outcomes in various cultural settings are found and hitherto no consensus can be made, we believe it will become more significant and promising to probe into cross-cultural similarities and differences in human perception and cognition, whereby the interactions among the self, others, and the environment can be better understood.

It should be noted, with a more contemporary focus, that our RT also indicates new directions and opportunities for scholars and practitioners to further develop cross-disciplinary studies at the intersection of cultural psychology and neuroscience in the post-pandemic world. More specifically, despite many people having encountered serious psychosocial challenges elicited by mandate quarantine and remote working or having experienced different levels of catastrophic cognitions during COVID-19, civilizations that refer to the broadest sense of cultural identity (Hungtington, 1993) seem to collide more frequently in this tough time and afterwards (Caputo et al., 2019; Chin et al., 2021). In such a vein, when facing increasing mental pressure, people have to cope with a greater variety of sensory changes in adapting to ongoing cultural changes all over the world. Thus, it is imperative to call for more research to delve into how culture is encoded in and decoded by the body and brain of people and to specify whether and how culture might be changed for the good of the whole.

## Author contributions

TC wrote the whole manuscript. C-LL collected the references and all published paper in this Research Topic. FC and FH provided guidance throughout the entire paper, helped with translation, and offered modification suggestions. All authors listed have made a substantial, direct, intellectual contribution to the work and approved it for publication.

## Funding

This paper was supported in part by the National Natural Science Foundation of China (No. 72272136), Zhejiang Provincial National Social Science Foundation (Nos. 20XXJC05Z and 21XXJC01ZD), Zhejiang Provincial Natural Science Foundation of China (No. LY20C090012), Humanities and Social Sciences Foundation of Ministry of Education of China (No. 18YJA840016), K.C. Wong Magna Fund in Ningbo University (RC190015 and RC202220), and College of Science and Technology Ningbo University (2022007).

## Conflict of interest

The authors declare that the research was conducted in the absence of any commercial or financial relationships that could be construed as a potential conflict of interest.

## Publisher's note

All claims expressed in this article are solely those of the authors and do not necessarily represent those of their affiliated organizations, or those of the publisher, the editors and the reviewers. Any product that may be evaluated in this article, or claim that may be made by its manufacturer, is not guaranteed or endorsed by the publisher.
